# Retraction: A Rare Case of Solitary Schwannoma of Submandibular Gland

**DOI:** 10.7759/cureus.r131

**Published:** 2024-01-25

**Authors:** Sultan O Gohal, Saud S Alradadi, Abdullah A Althomali, Abdulrahman A Alshehri, Razan A Zabarmawi, Abdullah J Taha, Sommaya A Ajabnoor, Haya A Al Azmi, Atheer I Alqubaysi, Mohammed A Alshamrani, Nasser F Almutair, Ibrahim M Alotaibi, Saad A Alotaibi, Ahmed M Abdullah, Faisal Al-Hawaj

**Affiliations:** 1 College of Medicine, Batterjee Medical College, Jeddah, SAU; 2 Dentistry, Vision Colleges, Riyadh, SAU; 3 College of Medicine, Taif University, Taif, SAU; 4 Medicine, King Saud bin Abdulaziz University for Health Sciences, Riyadh, SAU; 5 Medicine, Ibn Sina National College for Medical Studies, Jeddah, SAU; 6 Medicine, Dar Al Uloom University, Riyadh, SAU; 7 Medicine, King Khalid University, Abha, SAU; 8 Medicine, King Saud University, Riyadh, SAU; 9 Medicine, Shaqra University, Shaqra, SAU; 10 College of Medicine, Imam Abdulrahman Bin Faisal University, Dammam, SAU

The Editors-in-Chief have retracted this article. Concerns were raised regarding the identity of the authors on this article. Specifically, Faisal Alhaway and Malak Shammari have stated that they were added to this article without their knowledge or approval. The identity of the other authors could also not be verified. As the appropriate authorship of this work cannot be established, the Editors-in-Chief no longer have confidence in the results and conclusions of this article.

